# In Vivo Anticancer Activity of AZD3965: A Systematic Review

**DOI:** 10.3390/molecules27010181

**Published:** 2021-12-29

**Authors:** Ana Silva, Beatriz Antunes, Alberta Batista, Filipa Pinto-Ribeiro, Fátima Baltazar, Julieta Afonso

**Affiliations:** 1 Life and Health Sciences Research Institute (ICVS), School of Medicine, University of Minho, Campus de Gualtar, 4710-057 Braga, Portugal; anaisabelsilva2407@gmail.com (A.S.); a77084@alunos.uminho.pt (B.A.); alberta1997@gmail.com (A.B.); filiparibeiro@med.uminho.pt (F.P.-R.); 2 ICVS/3Bs-PT Government Associate Laboratory, 4805-017 Guimarães, Portugal

**Keywords:** cancer, glycolysis, lactate, monocarboxylate transporter 1, AZD3965, in vivo models

## Abstract

Proliferating cancer cells have high energy demands, which is mainly obtained through glycolysis. The transmembrane trafficking of lactate, a major metabolite produced by glycolytic cancer cells, relies on monocarboxylate transporters (MCTs). MCT1 optimally imports lactate, although it can work bidirectionally, and its activity has been linked to cancer aggressiveness and poor outcomes. AZD3965, a specific MCT1 inhibitor, was tested both in vitro and in vivo, with encouraging results; a phase I clinical trial has already been undertaken. Thus, analysis of the experimental evidence using AZD3965 in different cancer types could give valuable information for its clinical use. This systematic review aimed to assess the in vivo anticancer activity of AZD3965 either alone (monotherapy) or with other interventions (combination therapy). Study search was performed in nine different databases using the keywords “AZD3965 in vivo” as search terms. The results show that AZD3965 successfully decreased tumor growth and promoted intracellular lactate accumulation, which confirmed its effectiveness, especially in combined therapy. These results support the setup of clinical trials, but other important findings, namely AZD3965 enhanced activity when given in combination with other therapies, or MCT4-induced treatment resistance, should be further considered in the clinical trial design to improve therapy response.

## 1. Introduction

Cancer remains one of the biggest public health issues around the world. One of the many emerging therapeutic approaches is based on the metabolic particularities presented by cancer cells, mainly characterized by a switch from oxidative phosphorylation (OXPHOS) to accelerated glycolysis regardless of oxygen availability, resulting in intensive production of lactate [1]. To avoid cellular acidosis, cancer cells upregulate a series of pH regulators, including monocarboxylate transporters (MCTs) that extrude lactate to the tumor microenvironment (TME) via a proton-linked mechanism [2,3]. The overall profile of MCT overexpression in most tumors, herein focusing on MCT1, and its relation to aggressiveness and poor survival rates has launched several investigations aiming to identify viable inhibitors [4,5]. One of those inhibitors, AZD3965, has proven to be an effective and specific MCT1 inhibitor, which has already entered clinical trials in the UK [6]. In this review, we aimed to analyze the in vivo studies performed using AZD3965 in different types of cancer and infer the evidence of its pre-clinical effectiveness.

### 1.1. Altered Metabolism in Cancer and the Warburg Effect

**Figure 1:**
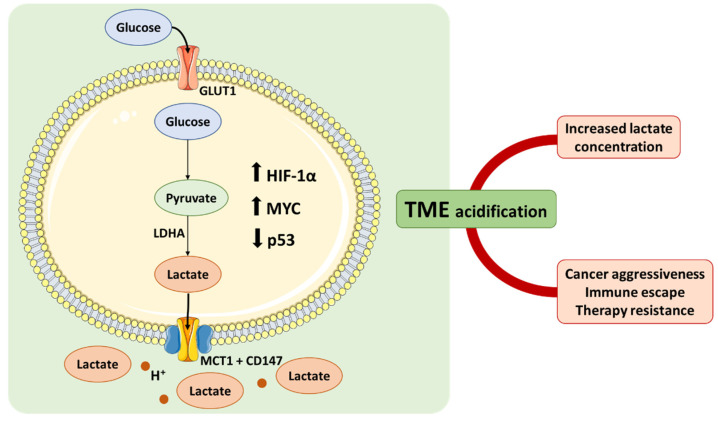
The Warburg effect and its implications on the tumor microenvironment. HIF-1α activation during hypoxic stress, oncogenic activation (e.g., *c-myc*), or loss of tumor suppressors (e.g., p53) leads to increased glucose consumption, acceleration of its metabolism, and increased lactate production. The consequent acidification of the TME promotes cancer aggressiveness, immune escape, and therapy resistance (GLUT1, glucose transporter 1; HIF-1α, hypoxia-inducible factor 1α; LDHA, lactate dehydrogenase A; MCT1, monocarboxylate transporter 1; TME, tumor microenvironment).

Malignant tumors are complex entities able to adjust their metabolism following their need to intensively proliferate, invade and metastasize, among other inherent characteristics that require high levels of energy [1]. Regardless of oxygen availability, cancer cells tend to preferably metabolize glucose to lactate via accelerated glycolysis, as observed by Otto Warburg in the 1920’s [1], instead of diverting the process to oxidative phosphorylation (OXPHOS), as happens in healthy, oxygenated cells. To compensate for the differences in adenosine triphosphate (ATP) production between the two processes, which is 18 times lower in glycolysis (2 ATP/mol of glucose in glycolysis vs. 32 ATP/mol in OXPHOS), Warburg observed that cancer cells consume copious amounts of glucose. This avidity for glucose is currently used for cancer diagnosis and staging, being the glucose analog ^18^fluoro-deoxyglucose (FDG) used in positron emission tomography (PET) scans [2,7]. Such an increased glucose consumption, accelerated metabolism, and accumulation of lactic acid is considered a hallmark of cancer and is currently named the “Warburg Effect” [2,8]. This metabolic switch confers an exceptional survival advantage to cancer cells and is thought to be triggered by the characteristics of the tumor microenvironment (TME) itself, including hypoxia and hypoxia-inducible factor 1 (HIF-1) transcription factor activity, as well as oncogenic activation [1] (Figure 1).

### 1.2. Lactate: From Metabolic Product to Signaling Agent

Malignant tumors uptake 47–70% of glucose from associated blood vessels, compared to 2–18% in normal tissues, and up to 66% of that glucose is converted to lactate, even in normoxic conditions [9]. Not surprisingly, lactate dehydrogenase isoform A (LDHA), an enzyme responsible for the transformation of pyruvate into lactate in the final step of anaerobic glycolysis, is upregulated in most malignancies [10]. LDHA expression is regulated by both *c-myc* oncogene and HIF-1 (Figure 1), and its increased activity reduces cells’ dependency on oxygen, as well as allowing for continuous glycolysis by regenerating NAD^+^ [10,11]. As a considerable amount of glucose is reduced to lactate, and to avoid cellular acidosis, lactate is exported from cells via MCTs, by a proton-coupled process. Accumulation of lactic acid and H^+^ in the TME leads to a significant decrease in pH, promoting immune escape, migration, and therapy resistance [12,13]. Lactate has also been suggested to play a role as a signaling agent both intra- and extracellularly. Intracellularly, lactate induces pseudohypoxia by HIF-1α stabilization and promotes angiogenic signaling by stimulating HIF-1α-mediated vascular endothelial growth factor (VEGF) expression. Extracellularly, lactate induces GPR81 activation in cancer cells, which has been reported to be linked to angiogenesis, proliferation (by, for instance, inducing MCT upregulation), enhanced DNA repair, and chemoresistance [14,15]. Not surprisingly, coupled with these important roles in tumor development, lactate has been associated with cancer aggressiveness, serving as a promising prognosis biomarker for some types of malignancies [16].

### 1.3. The Role of Monocarboxylate Transporters in Cancer Progression

As previously mentioned, the accumulation of lactate within cells leads to its efflux to avoid acidosis and allow the continuous glycolysis flux. Since lactate is a weak acid, at physiologic pH is negatively charged (deprotonated) and thus it requires a transporter to move it across the plasma membrane. MCTs are passive transporters encoded by the SCL16A solute carrier family of genes that comprises 14 members, although only the first 4 members (MCT1 to MCT4) are true monocarboxylate transporters. MCTs play an important role in many metabolic pathways as they facilitate the transport of monocarboxylates, short-chain fatty acids, and ketone bodies both through the plasma and mitochondrial membranes [17]. They have been given importance as prognostic markers in some cancers due to their common overexpression in most types of malignancies and their association with cancer aggressiveness [4]. Among the 14 MCT family members, MCTs 1 and 4 (SLC16A1 and SLC16A3, respectively), the most commonly upregulated isoforms in cancer, can either uptake lactate for energy purposes or export it to maintain homeostasis, and both have been linked to multidrug resistance as well as poor prognosis [18,19].

**Figure 2:**
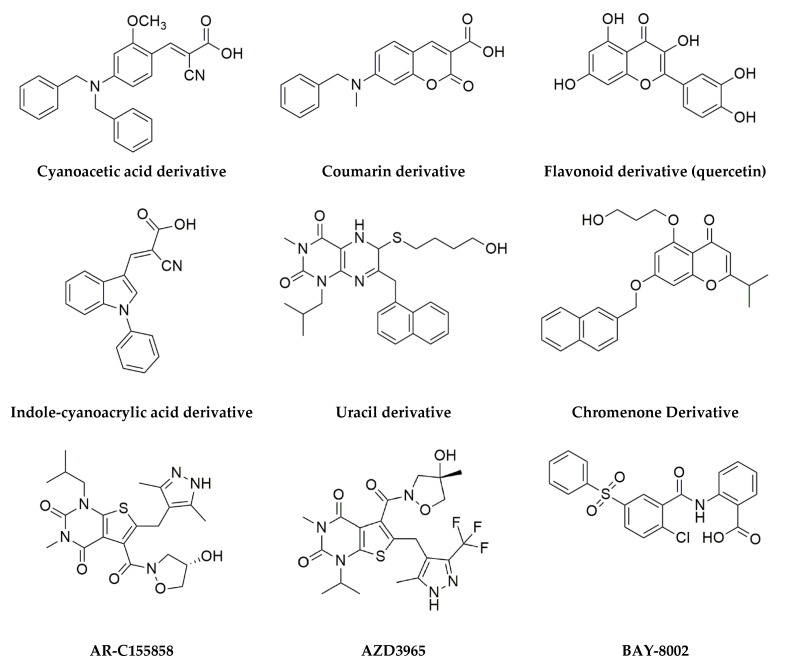
MCT1 inhibitors. Small molecules that belong to different chemical classes and for which MCT1 inhibitory activity has been described (reviewed in [18]). Cyanoacetic acid derivatives are dual MCT1/MCT4 inhibitors. AR-C155858 is a dual MCT1/MCT2 inhibitor, while AZD3965 partially inhibits MCT2 with a 6-fold lower affinity than MCT1. AZD3965 is the only compound that has entered the clinical trial phase.

MCT1, herein of particular interest, works as a proton-liked bidirectional lactate shuttle and was found to be upregulated in a variety of cancer types, including breast, head, and neck, bladder, colon, or glioblastoma, and to have an important role in regulating lactate exchange between cancer cells, control lactate signaling function and promoting metastasis [20]. Moreover, MCT1 was shown to be implicated in cisplatin-based therapy resistance in epithelial ovarian cancer and its knockdown inhibited tumor progression [4,20]. MCT1 is chaperoned by CD147 [21] and regulated by MYC [22] and p53 [23]. Due to the already referred role in cancer, MCT1 inhibition (Figure 2) stands as a therapeutic solution, and studies on this molecule as a therapeutic target revealed that its blockade improves therapy response and survival chances both in vitro and in vivo. MCT1 genetic knockdown by siRNAs impairs cell proliferation and induces apoptosis by increasing intracellular pH [24]. Additionally, MCT1 pharmacological inhibition has revealed promising results. A-cyano-4-hydroxycinnamate (CHC) has been tested for its antitumoral properties and has been shown to reduce lactate exchange, starve glucose-dependent cells, and promote necrosis, although it is not a specific MCT1 inhibitor [25]. The pharmaceutical company AstraZeneca has developed two drugs able to modulate MCT activity: AR-C155858, which targets MCTs 1 and 2, and AZD3965. This last compound is an MCT1 specific inhibitor that partially inhibits MCT2 with 6-fold lower affinity, and with no inhibitory activity for both MCT3 and MCT4 [25,26]. Recent studies, to be herein analyzed, have shown promising results regarding its antitumor activity [27,28].

### 1.4. AZ3965: A Specific MCT1 Inhibitor

AZD3965, a pyrrole pyrimidine derivate (Figure 2), is an oral bioavailable MCT1-specific inhibitor that has undergone a phase I clinical trial in the UK for advanced solid tumors and lymphomas. The primary outcome measure was to establish a biologically active and safe dose of AZD3965 for evaluation in future phase II clinical trials, being the determination of its pharmacokinetic profile in plasma and of objective tumor responses the secondary outcome measures of the trial [6]. A case report of refractory hyperlactaemic acidosis following the first dose of AZD3965 in a 47-year-old man with metastatic melanoma led to temporary recruitment suspension due to a possible drug-related event. However, the patient was diagnosed with “hyper-Warburgism”, a rare condition in which the high tumor burden is associated with a massive glucose uptake and lactate efflux from cancer cells. The clinical trial was then resumed, but screening of plasma lactate levels was added to the safety protocol, and elevated lactate levels were considered as exclusion criteria [29]. The first results showed that when treating advanced solid tumors, AZD3965 had dose-limiting toxicities above 20 mg (orally) regarding cardiac troponin rise and alterations in electroretinograms, but with overall good tolerability [30]. There were also small changes in lactate and ketone levels in the urine, which were attributed to the drug’s activity. Regarding lymphoma patients, the clinical trial showed the same effects on urine lactate and ketone concentration, good tolerability, and a noticeable reduction in FDG-PET in one patient [31].

Prior to the clinical trial, several studies showed AZD3965 efficiency as an antitumor agent in different types of cancer, both in vivo and in vitro. In small cell lung cancer, cells treated with the inhibitor showed improved radiosensitivity [32]. Additionally, in two other studies, the authors observed that treating cells with AZD3965 has better results in hypoxic rather than normoxic conditions; treated cells display significantly higher levels of lactate accumulation when compared to non-treated cells; AZD3965 upraised cell death mainly by necrosis and resistance to therapy occurred in cells displaying increased MCT4 expression [27,28]. MCT4 has been suggested as a key player in resistance to AZD3965 treatment, as it seems to create a compensatory mechanism able to cope with MCT1 blockade [33], a topic to be discussed further.

With this systematic review, we aimed to assess AZD3965 anticancer activity using in vivo models either alone or in combination. We searched nine databases in a selection process further to be described. A total of twelve eligible studies were screened for quality assessment.

## 2. Results

### 2.1. Literature Search

**Figure 3:**
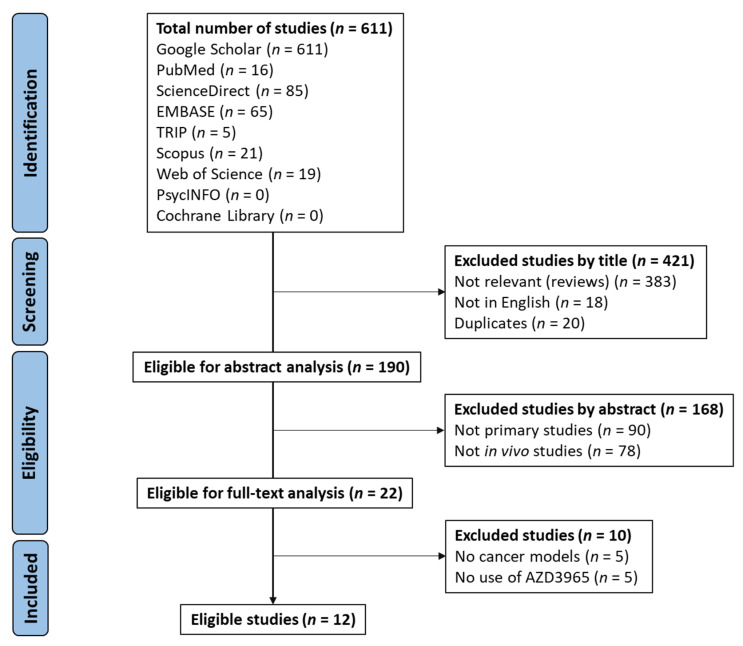
PRISMA 2020 flow diagram (adapted from [34]).

A literature search was conducted according to the Preferred Reporting Items for Systematic Reviews and Meta-Analyses (PRISMA) 2020 statement [34]. A total of 611 articles were first identified, as summarized in Figure 3. After screening, 190 studies were selected for abstract analysis, of which 168 were excluded, as these were not primary studies nor reported in in vivo experiments. The remaining studies (*n* = 22) were selected for full-text analysis, of which twelve studies were considered to comply with the selection criteria and included in this systematic review. The excluded studies contained no use of AZD3965 or its use to perform evaluations related to pharmacokinetics and testing of in vivo side effects, or no use of cancer animal models.

### 2.2. Characterization of the Studies

After a full analysis, twelve studies were considered to fit into the aim of assessing AZD3965 antitumor efficacy in vivo. Within those studies, two were performed using cell lines from small cell lung cancer [27,32], six used non-Hodgkin’s lymphoma cells [19,28,33,35,36,37], two used breast cancer cells [37,38], one used head and neck squamous cell carcinoma cells [39], one used lung squamous cell carcinoma cells [40], one used colorectal carcinoma cells [36] and one used renal cell carcinoma cells [41]. Studies reported administration of AZD3965 as monotherapy alone [19,27,35,36,37,38,41], or monotherapy and combined with either radiation (2Gy for 3 days) [32] or other pharmacological therapy—doxorubicin or rituximab [33], BAY-2243 [28], simvastatin [39] or JNJ-605 [40], all compared to vehicle control. AZD3965 was mainly administered via oral gavage in either 50 mg/kg or 100 mg/kg doses.

All studies used mouse models to test AZD3965. Regarding gender, two studies used male mice [27,41], one did not report gender [28], and the remaining used female mice. Animal age ranged from 4 to 14 weeks, although, on average, experiments were conducted in adult mice (over 8 weeks). Two publications [28,38] did not report animal age. The most commonly used strains were SCID and NSG and treatment was performed up to 43 days. Tumor induction was performed orthotopically in two studies [28,38] and non-orthotopically (subcutaneous injection) in the remaining studies [19,27,32,33,35,36,37,39,40,41]. Four to eleven animals were used per group, and groups treated with AZD3965 received the drug when tumor reached a volume between 100 and 500 mm^3^.

### 2.3. Global Quality

To evaluate the overall quality of the study, the ARRIVE Guidelines checklist for animal studies [42] were used. Studies were reviewed accordingly to indications provided by the guidelines and scored from 0 to 40. Final scores classified each study as general low quality (20 or lower points), moderate quality (21 to 30 points), and high quality (31 to 40 points). The large majority of the studies were classified as high quality (83.3%, 10 out of 12), with two moderate quality studies (16.7%). Out of all assessed aspects, mice housing and husbandry (item 9), sample size (item 10), baseline data (item 14), adverse events (item 17) and study limitations (item 18) were the parameters in which a more significant lack of information was noticed (Table 1).

### 2.4. Antitumoral Effectiveness of AZD3965 in In Vivo Models

Overall, reduction in tumor growth was considered the primary outcome (according to the main outcomes fixed for this review, as described in the Materials and Methods section) and was reported in all studies. Intratumor lactate concentration, a frequently reported secondary outcome, tended to increase significantly upon treatment (Table 2). Combined therapies detained better outcomes than monotherapies, as tumors were shown to be more sensitive to treatment when combining AZD3965 with either another pharmacological intervention or radiation.

## 3. Discussion

Cancer remains one of the leading causes of death worldwide. In 2020, cancer accounted for 19.1 million new cases and 10 million deaths [43], making cancer research crucial to improve treatment outcomes and patient life expectancy. The role of MCTs in cancer progression has been widely studied during the past years due to its link to aggressiveness and poor diagnosis, as mentioned above. Particularly, MCT1 has been intensively explored in many types of cancer for its role on lactate transport, in an attempt to disrupt lactate shuttling. Lactate efflux is known to contribute to the acidification of the TME and its levels are directly linked to poor prognosis, with increased angiogenesis, resistance to treatment, immune escape, and migration [12,13,44]. For these reasons, MCT1-specific inhibitors have been synthetized and tested in the cancer setting. This review collected and analyzed the existing information on in vivo testing of the MCT1 selective inhibitor AZD3965, which underwent a phase I clinical trial, to evaluate its safety as an anticancer agent. All of the studies included in this review described that treatment with AZD3965 promoted a significant reduction in tumor growth, accompanied by an increase in intratumor lactate concentrations, supporting its role in inhibiting MCT1 activity.

In recent years, many studies have tested the anticancer activity of AZD3965 in different types of cancer. The majority of these studies were performed in vitro and displayed promising results that justified in vivo cancer experiments (mice xenografts) to test its anticancer efficacy, pharmacokinetics, and possible side effects. Eligible publications selected for this systematic review reported in vitro experiments that corroborated the results obtained when the drug was tested in the mice models. Indeed, treatment with AZD3965 in vitro confirmed higher bidirectional lactate transport blockade, especially under hypoxic conditions [27,32]. Additionally, AZD3965 compromised cell proliferation in cell lines lacking MCT4 expression, contrary to what was seen in cells lines overexpressing MCT4. This fact, together with unaffected lactate transport, demonstrates that MCT4 engages in a compensatory method to prevent intracellular acidification, despite its lower affinity for lactate [27,28,36]. Beloueche-Babari et al. described that treatment with the MCT1 inhibitor was associated with increased mitochondrial metabolism and increase in TCA (tricarboxylic acid) cycle intermediates, enhancing ATP production and cell survival [35]. Interestingly, this was later contradicted by Braga et al. who obtained a small reduction in TCA intermediates in vivo, possibly resulting from PDH (pyruvate dehydrogenase) inhibition by PDK (pyruvate dehydrogenase kinase) due to intracellular pH variations [37].

In the presently selected studies, the authors performed experiments where AZD3965 was used as monotherapy [19,27,35,36,37,38,40], as monotherapy and combined with other therapies [28,32,33,39], and combined therapy exclusively [41], with a control group always included. After a full analysis of each publication, we evaluated the quality of the studies. We selected and analyzed the studies containing in vivo experiments that aimed to assess the anticancer properties of AZD3965 in any type of cancer. All selected studies were classified on overall quality using the ARRIVE Guidelines checklist [42], with a global end score of high quality. Out of all the parameters, animal information regarding housing and husbandry information, as well as sample size description within the methodology, was significantly lacking. Furthermore, studies limitedly reported baseline information about animal health and status before and throughout experiments, as well as describing and proposing ways to manage treatment-related adverse effects. Additionally, although results were discussed with clarity regarding the objectives of each experiment, most studies lack information about limitations with the animal models that they might have experienced, and their implications to future research. We also consider the lack of usage of patient-derived xenografts (PDX) to be an important limitation to selected studies. PDX experiments allow for better monitoring of the tumors’ specific intrinsic alterations, as well as allowing for predictability regarding therapeutic response to each type of tumor. Other limitations were the lack of MTD (maximum tolerated dosage) determination, critical for preclinical safety evaluation, and the fact that only immunocompromised mouse models were used, which restricts evaluation of the interaction between treatment and the immune system.

When used alone, with dosages of 50 or 100 mg/kg, AZD3965 was administered predominantly via oral gavage [19,27,28,32,33,35,36,38,39,40], but also via intraperitoneal or intravenous injection [37]. Results were compared with vehicle control groups and consistently reported a significant decrease in tumor growth and even tumor volume, indicating that progression was successfully slowed down. However, despite these results, tumor regression was not observed, possibly explained by AZD3965′s higher efficiency in hypoxic areas of the tumor [27]. All of the studies also reported increased intracellular lactate concentrations, confirming MCT1 blockade, but Beloueche-Babari et al. explained that this increase in lactate levels did not alter internal tumor pH, hypothesizing that this could be related to overexpressed pH regulators such as CAIX [35]. Braga et al. reported that despite increased tumor lactate concentrations, mice plasma lactate levels appeared not to have differences between treated and control groups [37]. Interestingly, some studies reported that after 24 h of treatment, intratumor lactate concentration was similar to baseline levels.

Performing mono- and combined therapy within the same study allowed for a clear comparison between strategies, leading the authors to conclude that combined therapy resulted in more accentuated outcomes regarding tumor growth and progression. Bola et al. verified that hypoxic/anoxic tumor cells became more susceptible to treatment and, therefore, they combined drug administration with radiation once radiation was shown to be less effective in hypoxic regions of the tumor [32]. According to the authors, radiotherapy has limitations on hypoxic cells, and the combination of both treatments resulted in a more accentuated decrease in tumor growth when compared to either treatment alone. Moreover, radiotherapy failed to promote intratumor lactate accumulation, while combined therapy resulted in this outcome [32]. Combination with rituximab, a monoclonal antibody that targets CD20 (a B cell antigen expressed during differentiation [45]), Curtis et al. reported tumor regression after 17 days of treatment and maintenance of this effect for 33 days after treatment cessation [33]. Similar results on the effects in tumor growth were obtained by two additional studies [28,39]. Guo et al. tested AZD3965 in KAT2A-overexpressing renal cell cancer xenografts. Inhibition of MCT1 with AZD3965 resulted in suppression of tumor growth induced by KAT2A (lysine acetyltransferase 2A, involved in the mediation of post-translational modification in histone H3 [46]) [41].

Besides the main results discussed above, other important aspects can be drawn out of these studies. In previous literature, resistance to treatment with AZD3965 was linked to overexpression of MCT4 [47]. Indeed, it was reported that resistance to treatment was coherent with MCT4 overexpression, suggesting that MCT4, although having a lower affinity for lactate [48], could be compensating and maintaining lactate exchanges in Burkitt’s lymphoma since it was not inhibited by AZD3965 [33]. In vitro acquired resistance by MCT4 overexpression was identified by six of the studies we selected herein [27,28,36,38,39,40]. We consider this to be worthy of close attention, and a limitation to most studies for the lack of data on MCT4’s influence on AZD3965 treatment outcomes, especially in vivo. AZD3965 was also reported to have an affinity for MCT2 and cells which overexpressed MCT2 could have contributed to treatment resistance [36]. It has been suggested that lactate export blockade affects glycolysis once it would result in a decrease of pyruvate and 2,3-biphosphoglicerate (2,3-GP) and an increase in glucose-6-phosphate (G6P) [20]. In Burkitt’s lymphoma, a slight decrease in pyruvate concentration was indeed observed, but it was back to baseline levels within 24 h. No changes in G6P or 2,3-GP were reported, meaning that intracellular lactate accumulation has a very limited effect on glycolysis [33].

Overall, the vast majority of eligible studies were considered to be of good quality and fit animal research requirements. AZD3965 successfully inhibited MCT1 and blocked lactate exchanges within tumors, being considered effective as an anticancer drug. All studies reported decreased tumor growth rates and a significant accumulation of intratumor lactate, with generally no implications in tumor regression. Despite these general positive outcomes, resistance promoted by MCT4 should stand as a crucial aspect to be taken into consideration in the setup of a clinical trial. Pre-clinical testing in non-immunocompromised mice seems also a relevant issue. MCTs are largely expressed in different tissues of the human body, making testing critical to understand which possible side effects treatment might have on healthy tissues. A very noticeable increase in radio- and chemosensitivity can stand out as one of the most important outcomes, as it opens the possibility to widen treatment options. We consider that the effectiveness of combined therapy should be taken into account in the design of future clinical trials, to improve treatment response. Most studies reported safety in the in vivo models when using combined therapy, which additionally stands out as an important clinical aspect. Nevertheless, a larger heterogeneity, with different cancer types being studied, preferably on PDX, is considered required, as different tumors might have different therapeutic responses.

## 4. Materials and Methods

### 4.1. Study Design, Aim, and Eligibility Criteria

This systematic review was conducted in accordance with the PRISMA 2020 Statement [34]; a review protocol was previously prepared, however, it was not registered in any registration database. The main aim of this study was to assess the antitumoral effectiveness of AZD3965 using in vivo models. Only articles including in vivo primary studies with cancer cells and comparative groups (to control vehicle) in which AZD3965 was used either in mono or combined therapy were included. Human studies, studies using AZD3965 but not experiments with cancer cells, grey literature, reviews, and studies not written in English or not available as full-text were not included in this systematic review. The presence of specific (main) outcomes was not considered an inclusion criterion. The main outcomes fixed for this systematic review were based on the background evidence, being divided into primary and secondary outcomes. The primary outcomes were tumor growth, tumor burden, growth rates after cessation of treatment and tolerability. The secondary outcomes were tumor lactate levels, intracellular tumor pH, changes in the glycolysis process, and ATP levels.

### 4.2. Search Strategy

To search articles to be included in this systematic review, PubMed, EMBASE, Science Direct, Scopus, Cochrane Library, Web of Science, TRIP, PsycINFO, and Google Scholar were used. The literature search was conducted from inception until the end of August 2021 using the keywords “AZD3965 in vivo” by 3 independent researchers (AS, BA, and AB). No sources other than the above-mentioned were searched and no authors were contacted.

### 4.3. Study Selection Strategy

Study selection was conducted by 3 independent researchers (AS, BA, and AB) with disagreements being resolved jointly by consensus and reviewed and approved by FB and JA. The screening was initially conducted by title and abstract and followed by full-text analysis. For each study, we extracted the following information: authors names, year of publication, country, methods (presence/absence of randomized groups, type of cancer, type of cancer cells, statistical analysis and outcome measure means), animal model (age, gender, number of animals per group), type of intervention, dosage and its frequency, route of administration, duration of each intervention, outcomes, and notes (funding, affiliation of AstraZeneca). These parameters were not used for comparisons between the studies but to compile existing information.

### 4.4. Quality Assessment and Data Synthesis

Analysis of quality was performed by AS and JA using the ARRIVE Guidelines Checklist—Animal Research: Reporting In vivo Experiments [42], which recommends checking a total of 20 parameters grouped by title, abstract, introduction (background, objectives), methods (ethical statement, study design, experimental procedures, experimental animals, housing and husbandry, sample size, allocating animals to experimental groups, experimental outcomes, and statistical methods), results (baseline data, numbers analyzed, outcomes and estimation, adverse events) and discussion (interpretation/scientific implications, generalisability/translation, funding). For each parameter, a score of 2 points was given to studies with the established requirements, 1 point was attributed if some aspect was lacking, and 0 points were attributed to studies completely lacking information. These results were translated into a color system: green dots for high-quality parameters, orange dots for moderate and red dots for low-quality parameters. In the end, a sum of all points was converted into a score corresponding to high-quality studies (≥31 points), moderate quality studies (21–30 points), or low-quality studies (≤20 points). Data from all included studies were organized in tables and a narrative description was performed.

## 5. Conclusions

AZD3965 effectiveness on tumor growth and disruption of lactate transport constitutes important evidence for its anti-cancer activity. Good tolerability in mice models, as well as an enhanced activity when combined with other therapeutic agents in solid tumors and lymphomas, support the rationale for the set-up of clinical trials. The main objectives of the launched phase I clinical trial were related to safety, namely determination of the maximum tolerated dose, potential side effects, and how they can be managed. However, considering the existing preclinical evidence, future trials should include a combination of AZD3965 with other therapeutic interventions. The possibility of resistance to treatment (namely MCT4 co-expression) should also be taken into consideration in the choice of cancer types, aiming to enhance clinical response. This review provides a compilation of pre-clinical information that could be useful in the design of future trials.

## Figures and Tables

**Table 1:** Quality assessment of the selected studies based on the ARRIVE Guidelines checklist for animal studies [42].

Reference	ARRIVE Guidelines Checklist																				Score	Classification
	1	2	3	4	5	6	7	8	9	10	11	12	13	14	15	16	17	18	19	20		
Polanski et al. 2014 [27]	●	●	●	●	●	●	●	●	●	●	●	●	●	●	●	●	●	●	●	●	36	**High**
Bola et al. 2014 [32]	●	●	●	●	●	●	●	●	●	●	●	●	●	●	●	●	●	●	●	●	34	**High**
Hong et al. 2016 [38]	●	●	●	●	●	●	●	●	●	●	●	●	●	●	●	●	●	●	●	●	22	**Moderate**
Noble et al. 2017 [28]	●	●	●	●	●	●	●	●	●	●	●	●	●	●	●	●	●	●	●	●	28	**Moderate**
Curtis et al. 2017 [33]	●	●	●	●	●	●	●	●	●	●	●	●	●	●	●	●	●	●	●	●	35	**High**
B.-Babari et al. 2017 [35]	●	●	●	●	●	●	●	●	●	●	●	●	●	●	●	●	●	●	●	●	35	**High**
Quanz et al. 2018 [36]	●	●	●	●	●	●	●	●	●	●	●	●	●	●	●	●	●	●	●	●	31	**High**
Mehibel et al. 2018 [39]	●	●	●	●	●	●	●	●	●	●	●	●	●	●	●	●	●	●	●	●	37	**High**
Apicella et al. 2018 [40]	●	●	●	●	●	●	●	●	●	●	●	●	●	●	●	●	●	●	●	●	38	**High**
B.-Babari et al. 2020 [19]	●	●	●	●	●	●	●	●	●	●	●	●	●	●	●	●	●	●	●	●	38	**High**
Braga et al. 2020 [37]	●	●	●	●	●	●	●	●	●	●	●	●	●	●	●	●	●	●	●	●	38	**High**
Guo et al. 2021 [41]	●	●	●	●	●	●	●	●	●	●	●	●	●	●	●	●	●	●	●	●	35	**High**

Quality of parameters: ● Low, 0 points; ● Moderate, 1 point; ● High, 2 points. Quality of study (score, classification): ≤20 points, low; 21–30 points, moderate; ≥31 points, high.

**Table 2:** Detailed information on eligible studies.

Reference	Strategy	Type of Cancer/ Cell Line	Mice Strain	Age (Weeks)	Sex	N	Dose	Outcomes
Polanski et al. 2014 [27]	Alone (og)	SCLC/COR-L103	NSG	8–14	male	6	100 mg/kg	↓ TG; ↑ ITL; no regression
Bola et al. 2014 [32]	Alone (og) and combined with radiotherapy	SCLC/H526	CD-1 nude	+8	female	8	100 mg/kg	↓ TG; ↑ ITL; ↑ radiosensitivity
Hong et al. 2016 [38]	Alone (og)	Breast cancer/SUM149PT	NSG	n.d.	female	n.d	0.1 ml/10 g	↓ TG; no FDG uptake changes
Noble et al. 2017 [28]	Alone and combined with BAY-2243 (og)	BL/CA46	NSG	n.d.	n.d.	8	100 mg/kg	↓ TG; ↑ ITL; ↑ chemosensitivity
Curtis et al. 2017 [33]	Alone (og) and combined with doxorubicin (iv) or rituximab (ip)	BL/Raji	SCID	8–12	female	11	50 or 100 mg/kg	↓ TG; ↑ ITL; ↑ chemosensitivity
B.-Babari et al. 2017 [35]	Alone (og)	BL/Raji	SCID	6–8	female	10	50 mg/kg	↓ TG; ↑ ITL
Quanz et al. 2018 [36]	Alone (og)	BL/Raji BL/Daudi DLBCL/WSU-DLCL2 CC/COLO320DM	NOD SCID; CB17 SCID; CB17 SCID; NMRI nu/nu	7–10	female	n.d.	50 mg/kg	↓ TG; ↑ ITL; ↓ ITP; no regression
Mehibel et al. 2018 [39]	Alone and combined with simvastatin (og)	HNSCC/ FaDu and CaL-27	CD-1 nude; SCID	8–14	female	28 (total)	100 mg/kg	↓ TG; ↑ chemosensitivity
Apicella et al. 2018 [40]	Alone and combined with JNJ-605 (og)	LSCC/RES-J EBC1	NOD SCID	6	female	9	100 mg/kg	↑ chemosensitivity
B.-Babari et al. 2020 [19]	Alone (og)	BL/Raji	SCID	6–8	female	9	50 mg/kg	↓ TG; ↓ TS
Braga et al. 2020 [37]	Alone (ip/iv)	DLBCL and breast cancer/U2932 and MDA-MB-231	NOD SCID; nu/nu-BALB/c	10–16	female	4–6	100 mg/kg	↓ TG; ↓ ITL; ↓ TCA cycle intermediates
Guo et al. 2021 [41]	Alone (ti)	RCC/A498 and Caki-2	nu/nu-BALB/c	4–6	male	5	n.s.	↓ TG; ↓ LM; ↓ KAT2A-induced progression

BL, Burkitt’s lymphoma; CC, colorectal carcinoma; DLBCL, diffuse large B-cell lymphoma; HNSCC, head and neck squamous cell carcinoma; ip, intraperitoneal injection; ITL, intra-tumor lactate concentration; ITP, intra-tumor pyruvate concentration; iv, intravenous injection; LSCC, lung squamous cell carcinoma; n.d., no data; n.s., not specified; og, oral gavage; RCC, renal cell carcinoma; SCLC, small cell lung cancer; TG, tumor growth; TCA, tricarboxylic acid; ti, tail injection; TS, tumor size; ↑, increased; ↓, decreased.
